# Research on the impact of regional integration policy on carbon emissions——Taking the Yangtze River Economic Belt as an example

**DOI:** 10.1371/journal.pone.0301968

**Published:** 2024-05-24

**Authors:** Yuxing Xu, Muhua Cui

**Affiliations:** School of Economics and Management, Huaibei Normal University, Huaibei, China; National University of Sciences and Technology, PAKISTAN

## Abstract

The Yangtze River Economic Belt serves as a paradigm of ecological integration and high-quality development within China. Under the constraints of the "Dual Carbon" goal, how does the integrative policy of the Yangtze River Economic Belt, aimed at reinforcing inter-regional industrial cooperation, impact carbon emissions across various provinces within the region? Leveraging panel data from 30 Chinese provinces spanning 2009–2019 and treating the 2016 promulgation of the "Yangtze River Economic Belt Development Planning Outline" as a quasi-natural experiment, this study employs a Difference-in-Differences (DID) model for discerning the effects of regional integration policies on carbon emissions, intensity, and efficiency in the 11 provinces of the Yangtze River Economic Belt. The research further delves into the underlying mechanisms through which policy interventions modulate provincial carbon emissions. Key findings include: (1) The policy’s enactment has significantly attenuated provincial carbon emissions and intensity, albeit somewhat hampering the enhancement of carbon efficiency—conclusions robust to parallel trend and placebo tests; (2) Heterogeneity analyses reveal disparities in the policy’s carbon emission effects, contingent on varying economic development levels and geographical loci; (3) Mechanistically, while the integration bolsters regional environmental governance and strengthens economic ties—thereby curtailing provincial emissions—it is evident that local governments have been somewhat inept in effectively channeling the influx of substantial short-term capital, hence stifling provincial carbon efficiency. In light of these insights, it is posited that local governments within the Yangtze River Economic Belt should ardently endeavor to refine and elevate regional industrial structures, champion the construction of an integrated regional market, intensify capital oversight and coordinated utilization, and enhance the efficiency of regional capital allocation, all in the quest to augment regional carbon emission reduction performance.

## 1. Introduction

In recent years, carbon emissions and the resultant climate change have drawn significant attention worldwide. As the globe’s preeminent carbon emitter, China faces the Herculean task of carbon reduction. Yet, embodying its role as a responsible emerging powerhouse, China, grounded in its national conditions and capabilities, has been ardently advocating energy conservation, emission reduction, and climate change mitigation over the past decade. Notably, during the 75th United Nations General Assembly on September 22, 2020, President Xi Jinping, representing China, solemnly pledged to the international community the nation’s aspirations to peak carbon emissions by 2030 and achieve carbon neutrality by 2060.

Situated in the heart of China, the Yangtze River Economic Belt, colloquially dubbed the "golden belt," extends across the nation’s eastern, central, and western territories, enveloping 11 provinces. It is a pioneer area for China’s regional economic integration and development and the fastest-growing region in China. It has become an essential engine for China’s economic growth. As of the end of 2022, the GDP of the Yangtze River Economic Belt reached 55.48 trillion yuan, accounting for 46.05% of the national total, and the population was 608 million, accounting for 43.07% of the national total. In addition, the forest coverage rate in the Yangtze River Basin reached 41.3%, and the area of rivers, lakes, reservoirs, and wetlands accounted for about 20% of the national total [1]. The Yangtze River Economic Belt is vital to the national ecology and economy [2]. However, due to the long-term development model of "heavy chemical industry" and "heavy coal" in the industrial and energy structures of the Yangtze River Economic Belt, the contradiction between resource environment and economic growth is prominent in the region. While the economy develops rapidly, the region’s ecological environment is also seriously threatened.

To reconcile the conspicuous discord between the resources, environment, and economic growth in the Yangtze River Economic Belt, the nation has laid paramount emphasis on its green development in recent years. China’s 14th Five-Year Plan distinctly propounds comprehensive promotion of the Yangtze River Economic Belt’s advancement, underpinning ecological primacy and green growth, synergistically advocating environmental preservation alongside economic development. General Secretary Xi Jinping has convened three symposiums, providing strategic directions for the Belt’s high-quality advancement, emphasizing that its development should prioritize ecological and green growth. The Belt is envisioned as the primary theater for green development, a pivotal conduit for domestic and international circulations, and the vanguard of high-quality economic progression.

Developing ecological civilization in the Yangtze River Economic Belt is a long-term and complex project. Despite the declining trend of energy consumption and carbon dioxide emission intensity per GDP in the whole basin [3], the industrial development mode of “heavy chemical industry” and “heavy coal” in the industrial structure and energy structure still exists [3]. Moreover, there are significant regional disparities in natural geography, resource endowment, energy structure, industrial structure, and development stage among different regions [4], highlighting the imbalance and insufficiency of low-carbon development in the Yangtze River Economic Belt. Therefore, under the “dual carbon” background, it is still an essential task for the current and future high-quality economic development of the Yangtze River Economic Belt to coordinate and promote the low-carbon transformation of the entire basin.

In the course of the Yangtze River Economic Belt’s integrated advancement, the imperative of curtailing excessive carbon emissions and championing green transition looms large for regional governments. As integration amplifies inter-regional industrial cooperation, what repercussions does it bear on the carbon emission dynamics of the constituent provinces? What underpins these effects? Delving into these intricacies, especially against the backdrop of the "Dual Carbon" goals, is of paramount importance. Addressing this conundrum is strategically vital for transforming the Yangtze River Economic Belt into a beacon of ecological integration and high-quality growth in China, resonating with the overarching "Dual Carbon" objectives.

## 2. Literature review

In contemporary scholarly discourse, the environmental ramifications of regional integration have primarily converged on two overarching avenues of investigation:

### 1. Environmental impact of regional integration

Much of the literature treats regional integration strategies as exogenous policy shocks. Techniques such as the Difference-in-Differences Model (DID) and the Synthetic Control Method (SCM) have been employed to assess the environmental outcomes of these integrative policies [5–7]. While studies have examined regional integration between various countries, such as the European Union’s enlargement [8, 9], others have looked at city-centric integration, for instance, between cities in the Pearl and Yangtze River Deltas [10, 11]. However, given the diverse research scopes and perspectives, no unified consensus on the environmental impact of regional integration has emerged. Chen et al. [12] postulate that the shared environmental regulations amongst EU member countries have led to significant environmental improvements post-integration. Zhao Lingdi et al. [6] argue that intensified economic ties in the Yangtze Delta post-integration have markedly increased the industrial wastewater discharge intensity across the city cluster. Gómez-Calvet et al. [13] discern that new EU member states exhibit the lowest energy efficiencies, suggesting substantial room for improvements in energy conservation and CO_2_ emission reductions.

### 2. Mechanisms underpinning the environmental effects of regional integration

Existing research contends that these environmental effects arise primarily from shifts in regional industrial structures and technological innovation. Lindmark M [14] and Paschem [15] suggest that post-integration industrial restructuring can lead to efficient resource allocation, thereby mitigating environmental degradation. Grossman [16] emphasizes the pivotal role of technological innovation in bolstering environmental quality, noting that advanced technologies often align more closely with sustainable economic growth. Within the domestic scholarly milieu, researchers such as Zheng Jun et al. [17] indicate that regional integration strategies facilitate the upgrade of regional industrial structures. Chen Xiqiang et al. [18] contend that regional integration can refine regional division-of-labor patterns, optimizing and adjusting the regional industrial structure. Deng Huihui [19] discovered that regional integration cooperation aids in dismantling administrative barriers, promoting the free flow of production factors on a broader scale, thereby leading to the upgrade of regional industries. Dong Chunfeng et al. [20] highlight that regional integration can enhance urban innovation capabilities through the synergistic guidance of governments and the market allocation effects of innovative elements. Shao Hanhua et al. [21] argue that the flow and integration effects of innovative resources brought about by regional integration, coupled with the spillovers of knowledge and technology and the enlargement of market scale, collectively bolster urban innovation. Ye Tanglin et al. [22] believe that regional integration can effectively elevate the spatial spillover effects in the innovation diffusion process.

### 3. Literature gap

To sum up, although the academic community has conducted extensive research on the effects of regional integration policies and achieved some noteworthy results, the existing literature mainly focuses on the environmental pollution problems caused by regional integration, such as wastewater and exhaust gas. It pays little attention to the impact of carbon emissions. Moreover, most existing studies only examine one aspect of carbon emissions, such as total amount, intensity, or efficiency. In addition, the current research also points out the critical role of regional cooperation in expanding market size [23], deepening economic ties, and facilitating goods circulation [24]. However, such cooperation may also bring severe environmental challenges while promoting regional economic development [25]. Especially in the absence of appropriate policies and legislation, regional economic cooperation may lead to increased carbon emissions [26], thus jeopardizing the sustainability of the environment. Therefore, technological innovation [27], technology transfer [28], and the widespread use of renewable energy [29] are significant. Based on these findings, we recognize the importance of promoting environmental sustainability through regional integration policies and need to explore more deeply the potential mechanisms of the specific impact of regional integration policies on carbon emissions.

Based on this, this paper takes the Yangtze River Economic Belt as an example and empirically analyzes the comprehensive impact of regional integration policies on carbon emissions from the perspectives of total amount, intensity, and efficiency and further infers and verifies the inherent mechanisms that cause these impacts. This study aims to fill the gap in the existing literature and provide a more comprehensive and in-depth perspective in order to understand the environmental impact of regional integration policies.

## 3. Mechanism analysis and research hypotheses

### 3.1 The mechanism of regional integration policies on carbon emissions in urban areas within the region

Regional integration fosters the interconnectivity of infrastructures between regions, thereby enhancing the ties among them and solidifying intergovernmental collaboration [30]. Consequently, the implementation of regional integration policies assists local governments in orchestrating large-scale intergovernmental coordinated governance, wherein collaborative defense against environmental pollution often becomes a pivotal facet of provincial collaboration. Such policies can effectively mitigate distortions in policy incentives stemming from disparities in environmental regulations, propelling the harmonization of environmental protection policies and oversight mechanisms on a broader scale [31]. The development facilitated by regional integration prompts uniformity in internal environmental regulations, leading to more stringent emission reduction measures. This is conducive to the strategic deployment and widespread propagation of low-carbon industries and technologies, thereby actualizing provincial low-carbon growth.

As the integration process intensifies, artificial barriers impeding the free flow of resources and factors between regions are progressively diminished, resulting in a pronounced acceleration toward market unification. With the consolidation of regional markets, there’s an amplification in the free movement of factors like labor and capital across a broader spectrum, tightening the economic links among regions. This will elevate the marginal cost of carbon dioxide emissions from both production and consumption perspectives [22], which in turn suppresses regional carbon emissions. Furthermore, regional integration hastens the free flow of goods and essential resources between areas, depressing factor prices and reducing corporate investment costs [32]. This encourages firms to redirect their focus towards technological innovation and performance management, thereby enhancing production efficiency and reducing carbon emissions.

**Fig 1:**
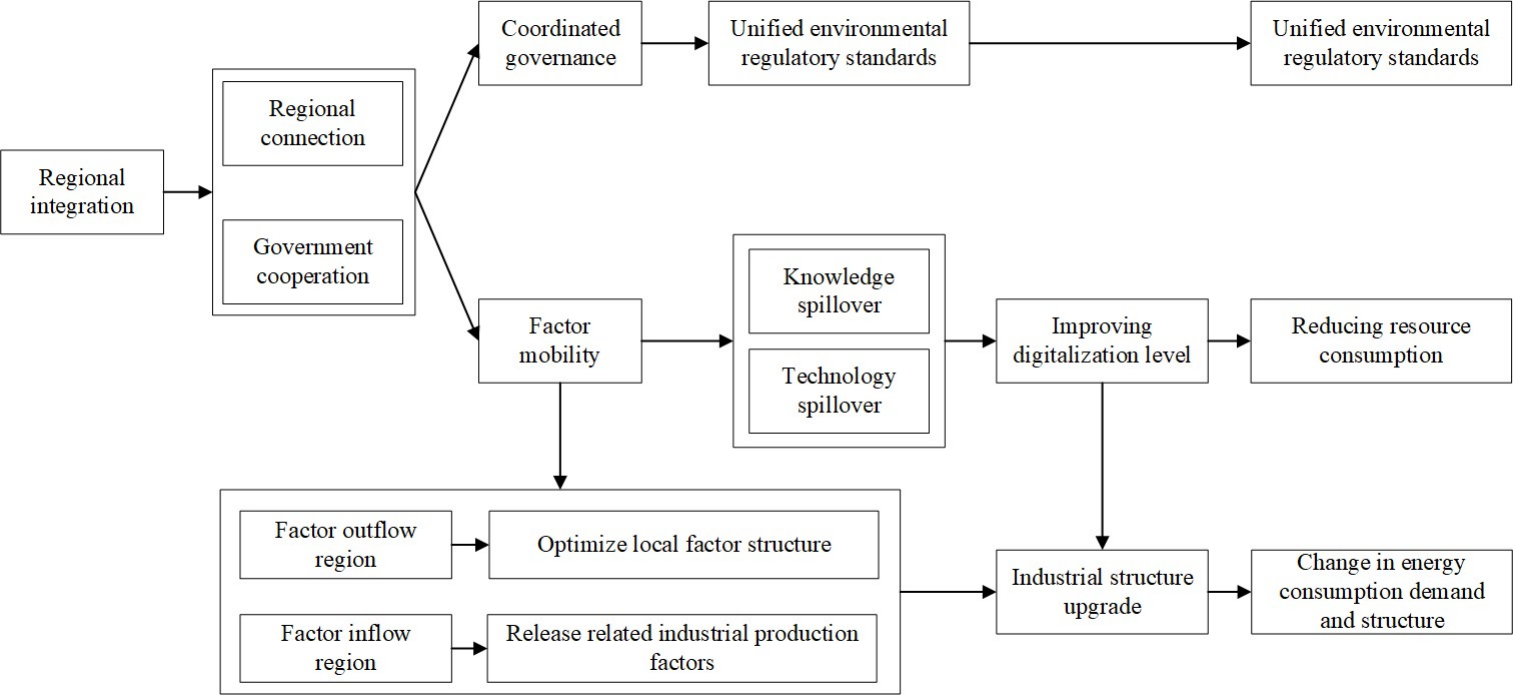
The carbon emission reduction path of regional integration policy.

Following regional integration, the ties between peripheral and central provinces strengthen. The free movement of resources catalyzes the restructuring and optimization of local industrial configurations, holistically advancing the industrial structural elevation of the Yangtze River Economic Belt. This not only impacts the regional industrial framework but also bolsters the diffusion of knowledge and technology spillovers, elevating the level of technological innovation in the region. Subsequently, this augments the digitalization level of the provinces; an upsurge in digital competency, in turn, promotes regional industrial digital transformation and the growth of digital industries, further propelling the industrial structural upgrade and mitigating carbon emissions [33]. Fig 1 delineates the pathway through which regional integration policies influence provincial carbon emission reduction.

Drawing upon the preceding analysis, this study posits its primary hypothesis:

Hypothesis H1: The integration policy of the Yangtze River Economic Belt can effectively reduce the carbon emissions of provinces.

### 3.2 Differential impacts of regional integration policies on carbon emissions within provinces

**Fig 2:**
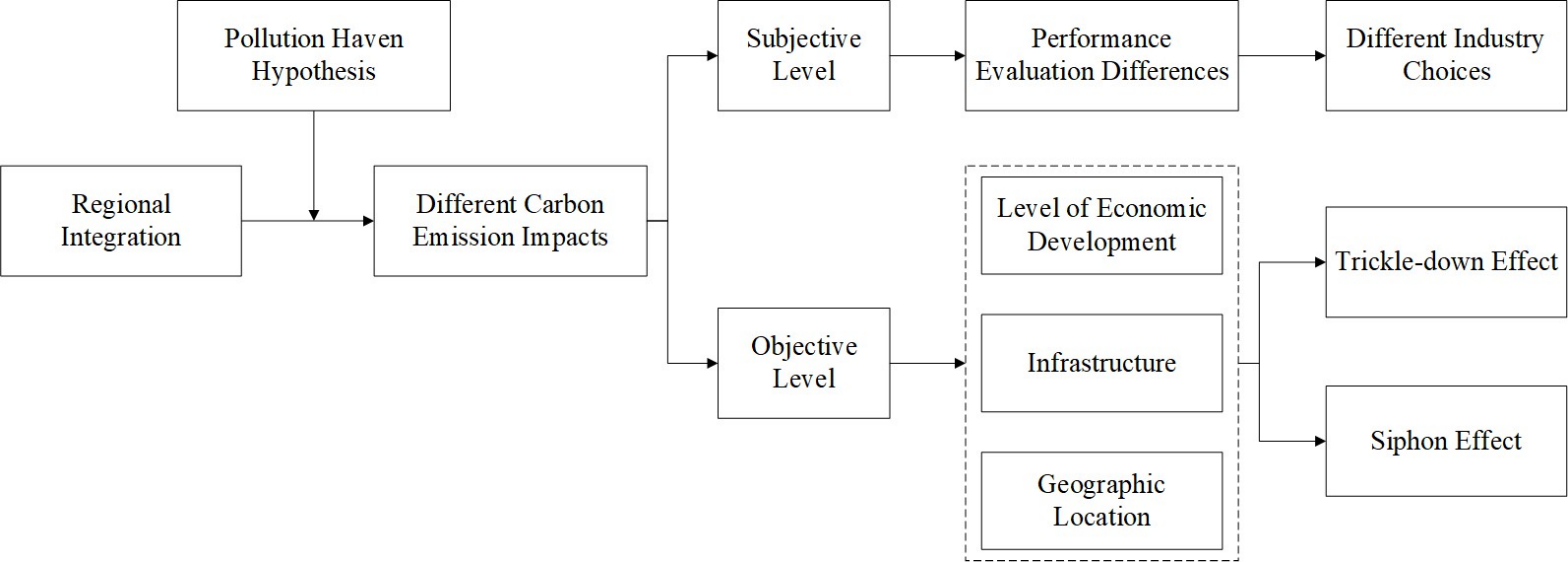
Heterogeneous pathways of regional integration policies on carbon emission reductions.

The "Pollution Haven Hypothesis" posits that during the advancement of international collaboration, less-developed regions may face the peril of pollution displacement from their more developed counterparts [34]. Consequently, the relocation of industries is selective, with regions possessing lenient environmental standards potentially becoming sanctuaries for pollution-intensive industries [35]. Studies indicate that pollution-intensive industries in provincial clusters exhibit a diffusion pattern from core to peripheral regions [36], suggesting that not all provinces reap developmental dividends from inter-regional collaboration. While regional integration bolsters economic ties between provinces, the distinct policy impacts on each province might lead to varied effects on carbon emissions. This can be elucidated from both subjective and objective perspectives:

Subjectively, during the integration process, due to differential performance assessments by higher-level governments across regions, local governments may develop varied preferences for economic and social welfare performances. This could result in core and peripheral provinces having disparate industrial selection predilections, consequently leading to non-uniform effects of regional integration policies on their carbon emissions. Objectively, the varying timelines when provinces join regional cooperation means that the carbon emission ramifications from integration policies exhibit regional disparities. As central provinces in the Yangtze River Economic Belt engaged in inter-regional collaboration earlier, integration facilitated these provinces to reap more "trickle-down" benefits, promptly fostering the upgrade of their industrial structures. In contrast, peripheral provinces, which joined the collaboration later and possessed varied levels of economic development, infrastructure, and geographical positions, might experience differentiated "trickle-down" effects, with some potentially still in the "polarization effect" phase. In sum, the variance in the carbon emissions impact of the integration policy on provinces within the region is an inevitable outcome of both subjective and objective factors. Fig 2 delineates the heterogeneous pathways of regional integration policies on carbon emission reductions.

Building on the aforementioned analysis, this paper proposes the second-layer hypothesis:

Hypothesis H2: The integration policies of the Yangtze River Economic Belt lead to heterogeneous impacts on carbon emissions across provinces.

## 4. Empirical analysis

### 4.1 Model selection

The academic community widely uses methods such as the Difference-in-Differences Model (DID) and the Synthetic Control Method (SCM) to evaluate the effects of policy interventions. SCM constructs a weighted average of multiple control units that matches the pre-intervention trend of the treated unit. However, it can only evaluate one treated unit at a time and requires a large sample size to obtain a good fit. DID, on the other hand, can evaluate multiple treated units simultaneously and allows for inherent differences across groups, as long as the control and treatment groups have the same trend in the absence of the intervention, thus identifying the causal effect of the policy. In this paper, we apply DID to study the impact of regional integration policies on urban carbon emissions. The baseline regression model is set as follows:

$$C_{it}=a_{0}+\alpha_{1}{DID}_{it}+\beta_{j}X_{it}+\delta_{t}+\gamma_{i}+\varepsilon_{it}$$ (1)

Where: *C*_*it*_ represents the dependent variables, which include carbon dioxide emissions, carbon dioxide emission intensity, and carbon dioxide emission efficiency. *X*_*it*_ is a vector of control variables for the respective model. *δ*_*t*_ and *γ*_*i*_ comprises time and individual fixed effects, respectively. *ε*_*it*_ is the error term. *DID* is a dummy variable for the regional integration policy. *α*_1_ is the regression coefficient, indicating the changes in carbon emissions brought about by the regional integration policy. *i* denotes the province, and *t* indicates the year.

### 4.2 Variables and data sources

#### 4.2.1 Dependent variables

*(1) Total carbon emissions*. Based on the national greenhouse gas inventory compiled according to the IPCC guidelines, this study selects the consumption of eight types of energy: raw coal, coke, crude oil, fuel oil, gasoline, kerosene, diesel, and natural gas. By integrating the carbon emissions calculation formula with the carbon emission coefficients, we estimate the carbon dioxide emissions of 30 provinces, municipalities, and autonomous regions in the country.

The carbon emissions calculation formula is as follows:

$${CO}_{2}=\sum_{i=1}^{8}C_{i}\times E_{i}$$ (2)

Where: *CO*_2_ represents the total carbon dioxide emissions. *E*_*i*_ is the consumption of the *i*th type of fossil fuel. *C*_*i*_ is the carbon emission coefficient for the *i*th type of fossil fuel.

Detailed data for each type of fossil fuel’s carbon emission coefficient is shown in **Table 1**.

**Table 1:** Carbon emission coefficients for various fossil fuels.

Energy Name	Carbon Emission Coefficient	Energy Name	Carbon Emission Coefficient
Raw Coal	1.9003	Fuel Oil	3.1705
Coke	2.8604	Diesel	3.0959
Gasoline	2.9251	Kerosene	3.0179
Crude Oil	3.0202	Natural Gas	2.1622

*(2) Carbon emission intensity*. Carbon emission intensity refers to the amount of carbon dioxide emissions per unit of GDP. This metric is primarily used to gauge the relationship between economic growth and the growth of carbon emissions. The formula for calculating carbon emission intensity is as follows:

$$CI=\frac{CO_{2}}{GDP}$$ (3)

Where: *CI* stands for carbon emission intensity. *GDP* denotes the Gross Domestic Product of the region. *CO*_2_ represents the total carbon dioxide emissions.

A lower value of *CI* implies that the region has managed to control its carbon emissions while achieving economic development, suggesting a higher quality of economic growth in that area. Conversely, a higher value indicates lower quality in economic development.

*(3) Carbon emission efficiency*. Carbon emission efficiency, a key indicator for measuring green economic development, aptly reflects the relationship between economic growth and carbon emissions through an input-output relation. Drawing inspiration from the study by Xu Bin et al. [37], we select regional fixed capital stock, the number of employed personnel at year-end, and total energy consumption as inputs. Provincial GDP is regarded as the desired output, while carbon dioxide emissions are considered as the undesired output. Utilizing the SBM-ML model for calculations, the carbon emission efficiency indicator system is illustrated in Table 2.

**Table 2:** Carbon emission efficiency indicator system.

First-Level Indicator	Second-Level Indicator	Indicator Description
Input Indicator	Labor Input	Number of employees per unit in each province over the years
	Capital Input	Represented by the actual capital stock of each province. Capital stock refers to the research of Zhang Jun et al. [38], calculated using the perpetual inventory method, the depreciation rate is set to 9.6%; the formula is: $K_{it}=K_{i,t-1}(1-\delta_{it})+I_{it}(4)$ Where, *K*_*it*_ represents the capital stock of province *i* in year *t*, this paper calculates it by converting the nominal value of fixed asset investment into actual value; *δ*_*it*_ is the depreciation rate, *I*_*it*_ is the capital flow.
	Energy Input	Total energy consumption calculated in standard coal equivalent
Desirable Output	Province GDP	GDP of the region calculated at constant 2008 prices
Undesirable Output	Province Carbon Emissions	Calculated based on the carbon emissions estimation method provided by the "IPCC National Greenhouse Gas Inventory Guidelines."

#### 4.2.2 Core explanatory variable

This study uses the issuance of the "Yangtze River Economic Belt Development Plan" in 2016 as a quasi-natural experiment, constructing a dummy variable for regional integration policy (DID) as the core explanatory variable. The assignment method for DID is as follows: for provinces incorporated within the scope of the "Development Plan" from 2016 onward, DID is set to 1; for provinces not covered by the "Development Plan," DID is set to 0.

#### 4.2.3 Control variables

Based on the STRIPAT model proposed by Dietz et al. [39], we selected our control variables. The standard form of the STRIPAT model is:

$$I=\alpha P^{a_{1}}A^{a_{2}}T^{a_{3}}e$$ (5)

Where: *I* represents the environmental impact. *α* is a constant term. *P* stands for population size. *A* denotes economic level. *T* is the technological level. *e* is the error term. *a*_1_, *a*_2_, *a*_3_ are the elasticity coefficients for population size, economic level, and technological level, respectively. Eq (5) indicates that population, technology, and economy are crucial factors impacting the environment. Hence, this paper initially controls for these three categories: provincial population density (POP) represents the scale of the population; the sum of the provincial utility model patent authorizations and invention patent authorizations (PATENT) characterizes the level of provincial innovation; and the provincial per capita gross product (PGDP) represents economic variables. Additionally, drawing upon existing literature [40], this study incorporates factors such as the level of openness to foreign trade, environmental regulation, infrastructure quality, human capital, and urbanization into the analytical model. The specific definitions of each variable are outlined in Table 3.

**Table 3:** Measurement of control variables.

Variable Name	Variable Symbol	Variable Measure
Level of Opening to the Outside World	FDI	Represented by the proportion of the amount of foreign capital used in each province in the current year to GDP. The amount of foreign capital used is converted into RMB according to the average exchange rate of RMB to the US dollar in the current year.
Environmental Regulation	ER	Measured by the ratio of industrial pollution control investment to GDP
Infrastructure Level	INF	Represented by the per capita paved road area
Government Intervention Level	INTER	Represented by the ratio of local fiscal general budget expenditure to GDP
Human Capital Level	HUMAN	Measured by the ratio of the number of undergraduate students in general higher education institutions to the permanent population
Urbanization Level	URBAN	Represented by the urbanization rate of the permanent population

#### 4.2.4 Data sources and descriptive statistics

The data utilized in this study primarily stems from the "China Statistical Yearbook" spanning the years 2010–2020. Energy consumption data is sourced from the "China Energy Statistical Yearbook" for the years 2010–2020, while data concerning the investment in industrial pollution control is derived from the "China Environmental Statistical Yearbook" from 2010 to 2020. The average low calorific value of various energy types is based on the "Comprehensive Energy Consumption Calculation Guidelines." The carbon content per unit calorific value and carbon oxidation factors of various energy sources are taken from the "Provincial Greenhouse Gas Inventory Compilation Guide." Descriptive statistics for each variable can be found in Table 4.

**Table 4:** Descriptive statistics table.

Variable	Sample Size	Mean	Standard Deviation	Min	Max
CO_2_	330	398.29	282.75	36.59	1471.31
CI	330	2588.21	1921.16	216.12	8779.53
ML	330	0.97	0.04	0.81	1.14
DID	330	0.13	0.34	0.00	1.00
PDGP	330	47803.62	25829.69	10814.00	161776.00
POP	330	2843.84	1171.00	764.00	5821.00
PATENT	330	32791.28	47440.11	124.00	342483.00
HUMAN	330	1.17	0.44	0.47	2.60
INF	330	15.05	4.67	4.04	26.20
URBAN	330	57.04	12.90	29.89	89.60
FDI	330	2.26	2.10	0.01	15.02
ER	330	0.13	0.12	0.002	1.10
INTER	330	25.81	11.31	10.98	75.83

### 4.3 Empirical results analysis

#### 4.3.1 Baseline regression results

This study designates the 11 provinces of the Yangtze River Economic Belt as the treatment group, while the other 19 provinces, municipalities, and autonomous regions across the nation, excluding Tibet, serve as the control group. We employ the Difference-in-Differences model to test the net effect of regional integration policies on provincial carbon emissions. The regression results are presented in Table 5. In Table 5, models (1)(2), (3)(4), and (5)(6) pertain to total carbon emissions, intensity, and efficiency, respectively.

**Table 5:** Estimated results of the baseline model.

	(1)	(2)	(3)	(4)	(5)	(6)
DID	-47.759***	-55.816***	-50.037	-95.568	-0.025***	-0.020***
	(-4.42)	(-4.50)	(-0.69)	(-1.58)	(-4.58)	(-3.34)
Control Variables	N	Y	N	Y	N	Y
Time FE	Y	Y	Y	Y	Y	Y
Province FE	Y	Y	Y	Y	Y	Y
N	330	330	330	330	330	330
R^2^	0.972	0.975	0.980	0.984	0.562	0.628
Adj.R^2^	0.969	0.971	0.977	0.982	0.502	0.563

Note: The values in parentheses are t values, ***, **, and * represent significance at the 1%, 5%, and 10% levels, respectively, the same as below.

From the regression results of models (1), (2) and models (5), (6), regardless of whether control variables are included, the coefficients of the regional integration policy are significant at the 1% level and negative. This suggests that while the integration policy has promoted carbon emission reductions across the Yangtze River Economic Belt, it has also suppressed improvements in carbon emission efficiency. The potential reasons behind this are twofold: On one hand, the integration policy has facilitated intra-regional factor mobility, optimizing the industrial structure, which in turn reduces carbon emissions. On the other hand, the integration policy has led to a substantial influx of resources into the Yangtze River Economic Belt. However, local governments have been unable to allocate these resources effectively in a short time frame, thereby impacting the improvement of carbon emission efficiency. The estimates of models (3) and (4) are negative but insignificant, suggesting that the integration policy does not significantly reduce carbon emission intensity. One possible explanation is that the policy has been implemented for a short period of time, and the regions are still adapting to the regional coordination mechanism, which attenuates the policy’s impact on carbon emission intensity.

#### 4.3.2 Parallel trends test

A crucial premise of employing the Difference-in-Differences method is that the treatment group and control group must satisfy the parallel trends assumption prior to the policy implementation. If differences in time trends existed between the two groups before the policy shock, changes in carbon emissions might not be attributable to the policy but rather to pre-existing temporal trends. To address this, our study utilizes an event study framework to examine whether carbon emissions of the treatment and control groups followed parallel trends before the issuance of the "Yangtze River Economic Belt Development Plan Outline." The following econometric model is constructed for testing:

$$C_{it}=\partial_{0}+\sum_{2009}^{2019}\beta_{\gamma }{DID}_{it}+\eta X_{it}+\lambda_{i}+\gamma_{t}+\varepsilon_{it}$$ (6)

**Fig 3:**
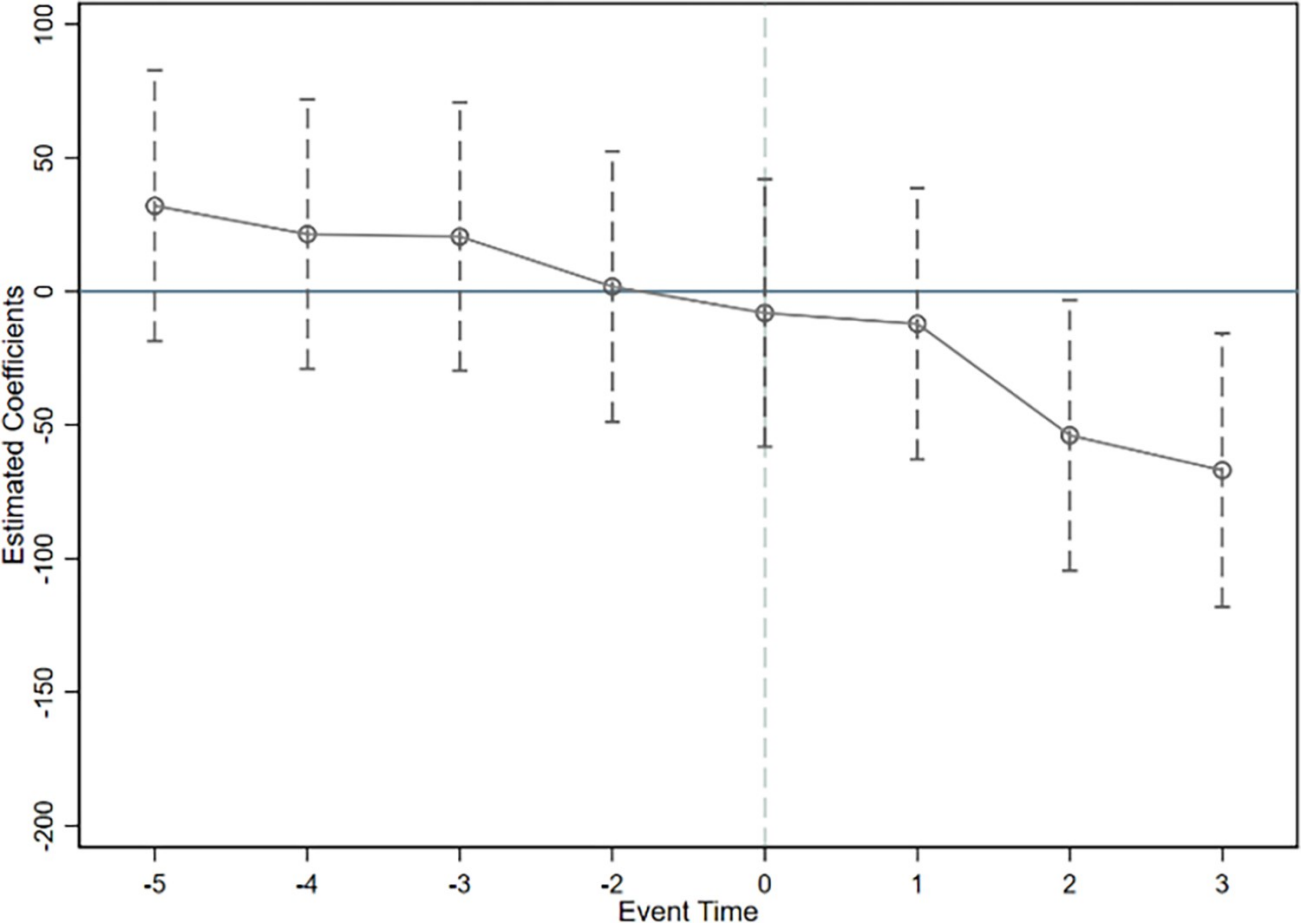
Parallel trend test of total carbon emissions.

**Fig 4:**
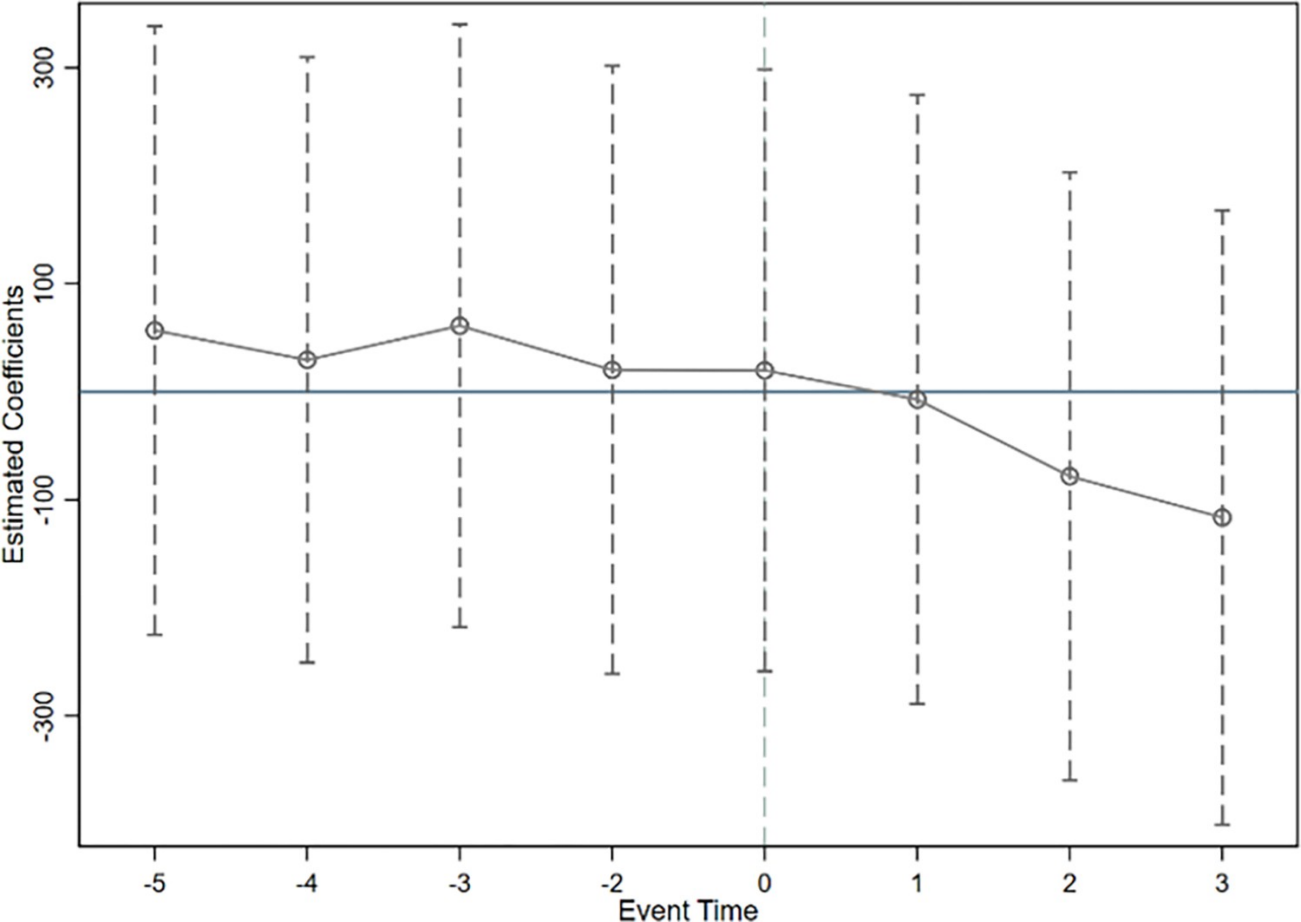
Parallel trend test of carbon emission intensity.

**Fig 5:**
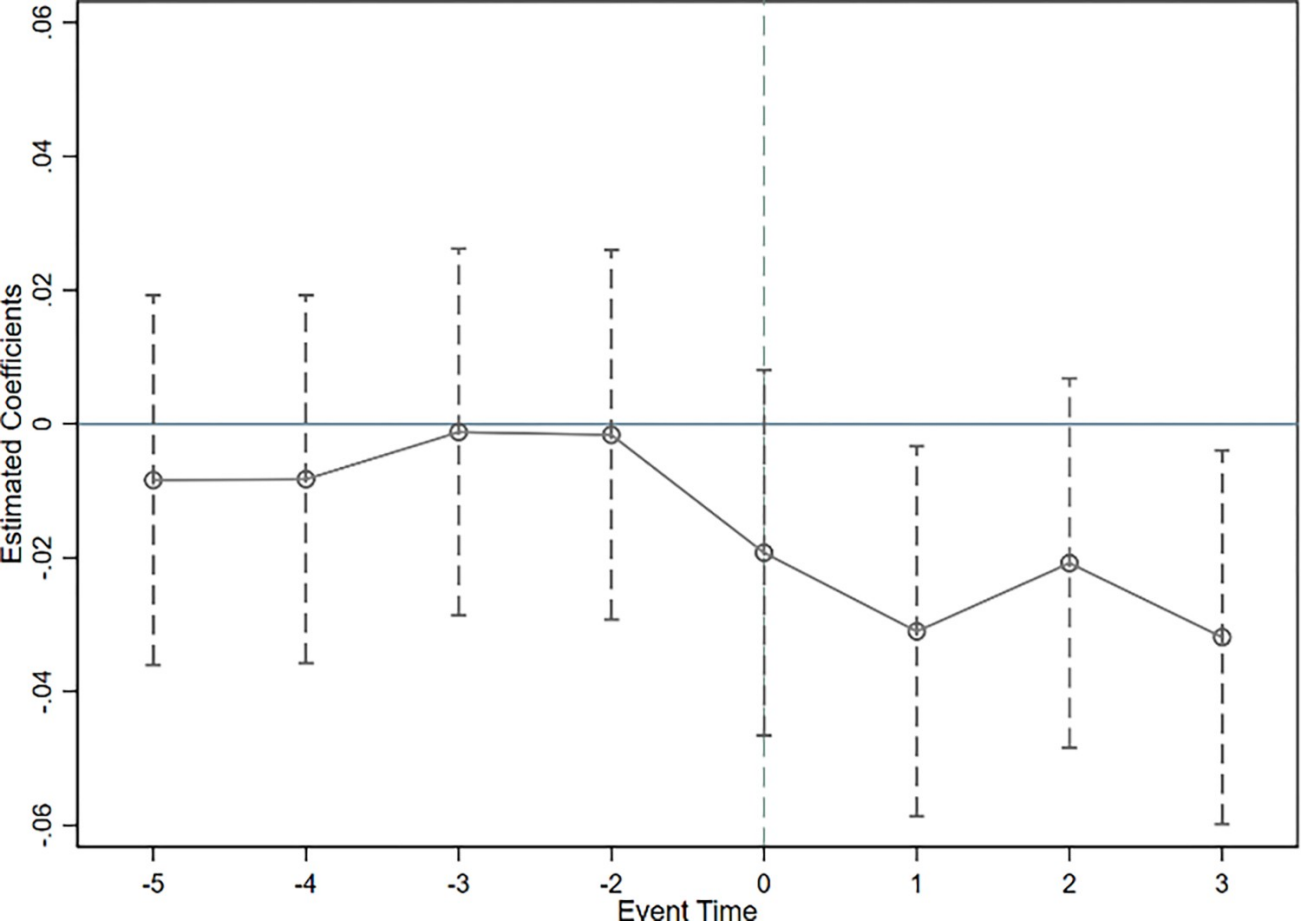
Parallel trend test of carbon emission efficiency.

Where *C*_*it*_ represents the dependent variable, that is, the total carbon emissions, intensity, and efficiency. *DID*_*it*_ represents the dummy variable of the year when the regional integration policy was introduced. *β*_*γ*_ represents the impact on provincial carbon emissions in various periods following the introduction of the regional integration policy. In the specific regressions, this study uses the year prior to the implementation of the regional integration policy as the base period. Figs 3–5 respectively report the estimated parameter values for total carbon emissions, intensity, and efficiency, along with their corresponding 95% confidence intervals.

Figs 3 to 5 indicate that before the implementation of the integration policy, the confidence intervals of the estimated coefficients for the dummy variable of each year all encompass 0, suggesting they do not pass the significance level of 5%. This validates that there was no significant difference in carbon emissions between the treatment group and the control group before the policy was implemented, thereby meeting the parallel trends assumption. Consequently, the change in carbon emissions in the treatment group after the integration policy was due to the policy effect and not because of pre-existing differences.

### 4.4 Robustness check

#### (1) Placebo test

**Fig 6:**
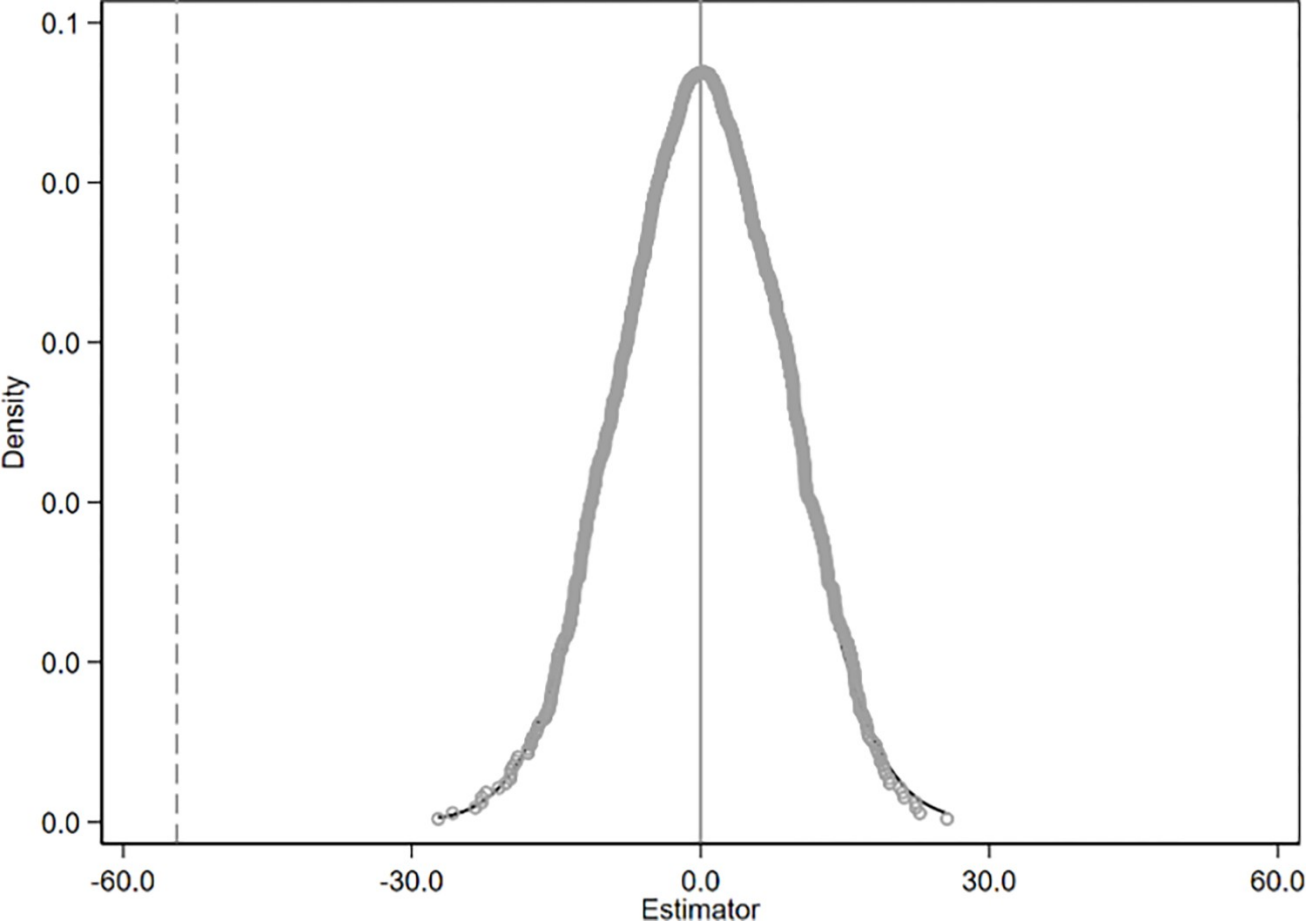
Placebo test results of total carbon emissions.

**Fig 7:**
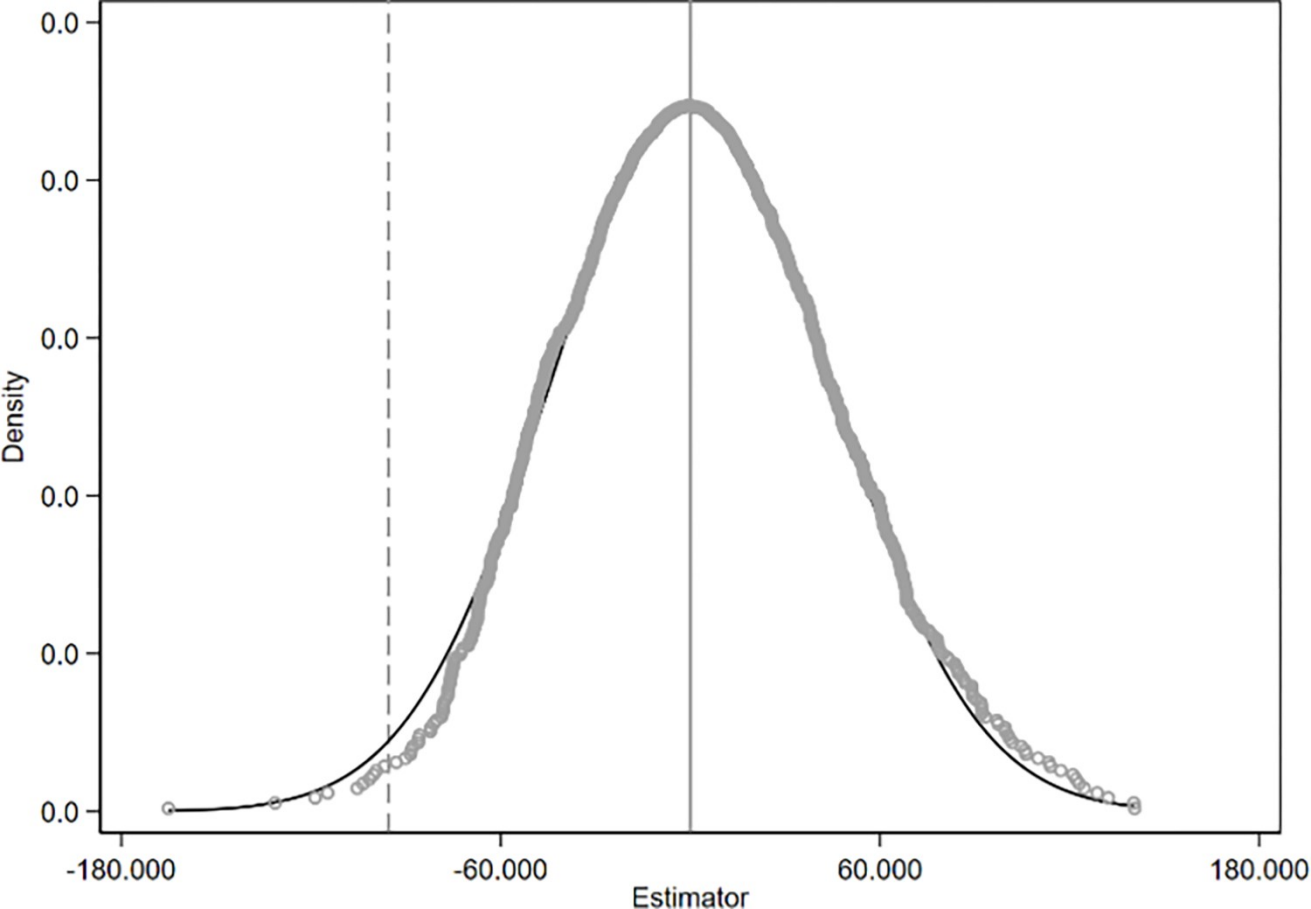
Placebo test results of carbon emission intensity.

**Fig 8:**
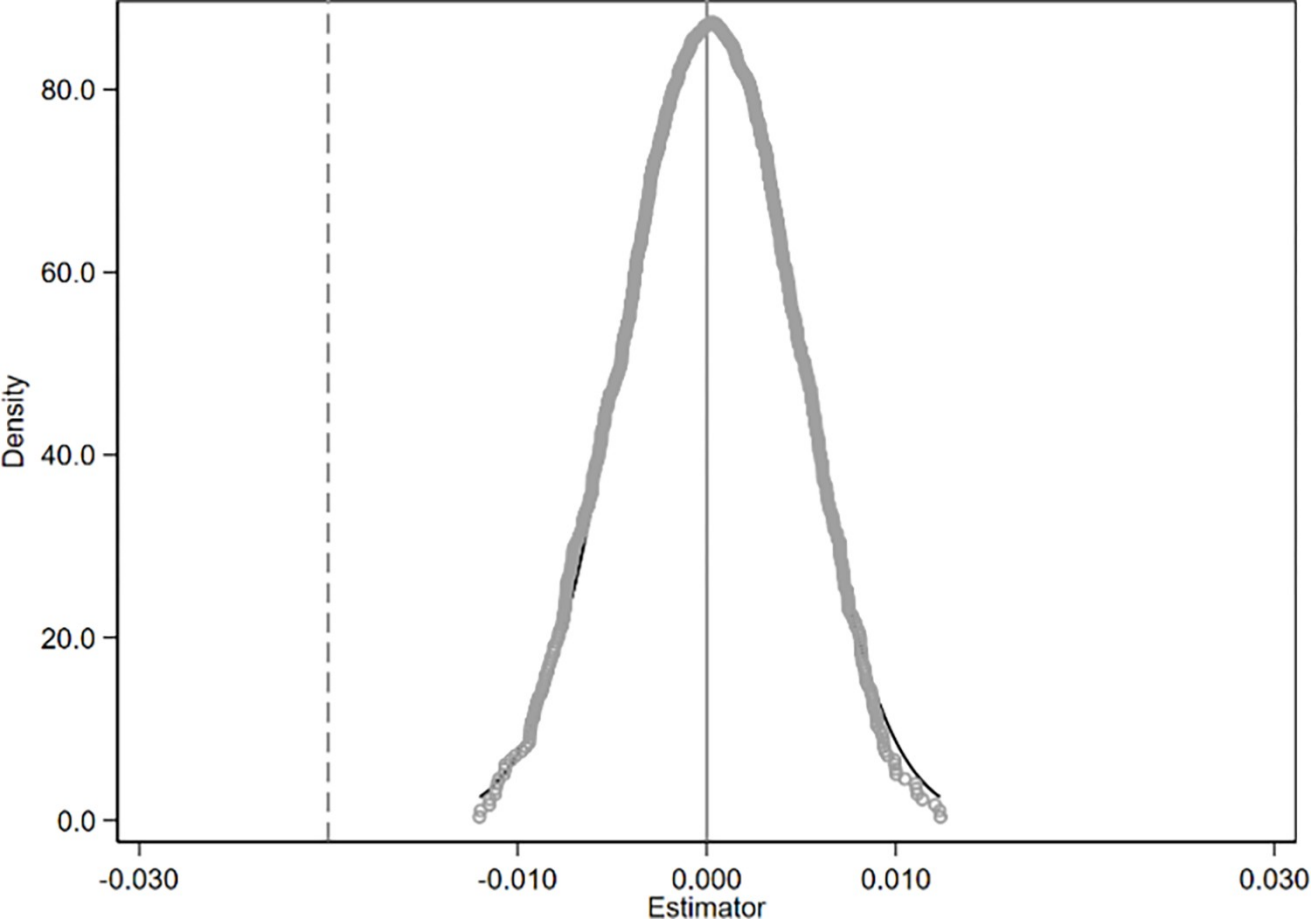
Placebo test results of carbon emission efficiency.

This study employs a counterfactual approach for the placebo test to rule out issues related to omitted variables and the potential randomness of the policy. Specifically, a sham experiment is constructed by randomly designating treatment groups and policy implementation times, and regressions are conducted to obtain the estimated coefficients of the spurious effects. After repeating the sham experiment 1,000 times, we plotted the distribution of the DID estimated coefficients (as shown in Figs 6–8). If the coefficients of the spurious effects exhibit a symmetric inverted U-shaped distribution centered around 0, it would indicate that the carbon emission effect in the base analysis is indeed caused by the integration policy.

Figs 6–8 present the distribution plots of the estimated coefficients. The dashed line in the figures represents the true policy coefficient. The results demonstrate that the estimated coefficients of the false difference-in-differences (DID) terms are uniformly distributed around zero and exhibit a symmetric inverted U-shaped distribution. This is significantly different from the true coefficient, indicating that the effects on provincial carbon emissions are primarily driven by the regional integration policy, and the core conclusion remains robust.

#### (2) Excluding interference from relevant policies

In addition to the influence of the integration policy, carbon emissions in each province of the Yangtze River Economic Belt could potentially be affected by other policies. To control for this, based on the "Notification on Launching the Carbon Emission Trading Pilot Program," this study excludes the samples of seven provinces related to the carbon trading pilot policy, namely Beijing, Tianjin, Shanghai, Chongqing, Guangdong, Hubei, and Fujian, and conducts a regression on the new sample. As shown in Table 6, the DID coefficients for total carbon emissions and efficiency are still significantly negative, consistent with the baseline regression.

**Table 6:** Regression results after excluding related policies.

	(1)	(2)	(3)	(4)	(5)	(6)
DID	-53.319***	-90.480***	-34.785	-126.842	-0.028***	-0.025***
	(-4.02)	(-5.36)	(-0.37)	(-1.45)	(-4.52)	(-3.50)
Control Variables	N	Y	N	Y	N	Y
Time FE	Y	Y	Y	Y	Y	Y
Province FE	Y	Y	Y	Y	Y	Y
N	253	253	253	253	253	253
R^2^	0.972	0.977	0.978	0.982	0.574	0.646
Adj.R^2^	0.968	0.972	0.975	0.979	0.509	0.575

## 5. Examination of the impact mechanism

Given the previous findings, regional integration indeed significantly promotes the carbon reduction effect in the Yangtze River Economic Belt. However, it also reduces the carbon emission efficiency of the belt. How exactly does this influence come into play? In the subsequent sections, a comprehensive analysis of the underlying mechanism of this impact will be presented.

### 5.1 Mechanism of regional integration policy in promoting carbon reduction

Based on the impact mechanisms and hypotheses presented earlier, as the regional integration process accelerates, various artificial factors hindering inter-regional factor flows will gradually weaken or be eliminated, and cooperation among regional governments will become more closely knit. For the Yangtze River Economic Belt, within the context of emphasizing "joint major protection without major development," areas along the belt will prioritize ecological and environmental protection. Therefore, collaborative environmental governance becomes a crucial prerequisite for deepening cooperation. This study first examines the impact of the integration policy on regional environmental collaborative governance. It then investigates whether the integration policy has driven market consolidation, enhanced the region’s digitalization level, and thereby promoted the optimization and upgrading of provincial industrial structures.

#### 1. Deepening collaborative governance: Reducing environmental decentralization

To measure the variability in environmental regulatory intensity, this study draws inspiration from the method proposed by Bai Junhong et al. [41]. We adopt environmental decentralization to characterize the deepening mechanism of collaborative governance. The calculation method for environmental decentralization is illustrated as follows in Eq (7):

$${ED}_{it}=\frac{{LESP}_{it}/{POP}_{it}}{{NESP}_{t}/{POP}_{t}}\left[1-({GDP}_{it}/{GDP}_{t}\right)]$$ (7)

Wherein: *i* denotes the province. *t* denotes the year. *ED*_*it*_ represents the degree of environmental decentralization in each province. *LESP*_*it*_ signifies the number of province employees in the environmental protection sector for each province. *POP*_*it*_ denotes the total population of each province at the end of the year. *NESP*_*t*_ signifies the number of province employees in the environmental protection sector in China in *t* year. *POP*_*t*_ denotes the total population of China at the end of *t* year. 1−(*GDP*_*it*_/*GDP*_*t*_) is an economic scaling factor, which can eliminate the interference of economic scale on the actual degree of environmental decentralization. The larger the value of *ED*_*it*_, the higher the degree of environmental decentralization.

To investigate whether the integration policy can influence the unified environmental regulatory level of the region, the dependent variable in Eq (1) is replaced with the environmental decentralization variable, and a regression analysis is conducted. The results are presented in Table 8, Model (1). The regression outcome reveals that the DID coefficient is significantly negative. This indicates that the implementation of the regional integration policy can establish unified environmental regulatory rules, thereby reducing the degree of environmental decentralization.

Carbon emissions, as public goods with negative externalities, necessitate deepened regional collaborative governance for carbon reduction [42]. The decline in environmental decentralization means that local governments no longer selectively execute environmental policies. This weakens the policy distortions brought about by environmental system differences, conducive to realizing stricter institutional constraints and more significant emission reductions at a lower cost [31], thereby generating an overall carbon reduction effect in the Yangtze River Economic Belt [43]. In general, the regional integration policy, by establishing a unified environmental regulatory mechanism, has reduced regional environmental regulatory disparities, influencing the regional carbon emission levels.

### 5.2 Promotion of market integration: Strengthening of economic ties across regions

The regional integration policy has reduced the degree of environmental decentralization among provinces, indirectly reflecting tighter cooperation between local governments [44]. This is conducive to eliminating man-made barriers that hinder factor mobility between regions, accelerating inter-regional factor flow and division of labor, promoting market integration, and strengthening regional economic ties.

To this end, this study uses economic ties to represent the market integration mechanism. Drawing on the method of Liu Naiquan et al. [45], a modified gravity model is adopted to measure the economic relations of provinces in a given year:

$$BC_{i,t}=\sum_{j=29}\;EC_{ij,t}$$ (8)

$$EC_{ij,t}=W_{ij,t}\cdot \sqrt{P_{i,t}\cdot GDP_{j,t}}\cdot \sqrt{P_{j,t}\cdot GDP_{j,t}}/D_{i,j}^{2}$$ (9)

$$W_{ij,t}=GDP_{i,t}/(GDP_{i,t}+GDP_{j,t})$$ (10)

Wherein: *EC*_*i*,*j*,*t*_ represents the economic relationship strength between provinces i and j. *P*_*i*,*t*_ and *P*_*j*,*t*_ respectively denote the population of provinces i and j. *GDP*_*i*,*t*_ and *GDP*_*j*,*t*_ respectively signify the GDP of provinces i and j. *D*_*i*,*j*_ represents the distance between provinces i and j. The larger the value of *D*_*i*,*j*_, the stronger the regional economic ties.

To test whether the integration policy can influence regional market integration, the dependent variable in Eq (1) is replaced with the economic relationship variable for regression. The results, as shown in Table 8 Model (2), reveal that the DID coefficient is significantly positive. This suggests that the integration policy facilitates breaking down administrative divisions and market barriers, promoting market unification and tighter regional economic ties, and spurring the orderly free flow of factors.

The orderly free movement of factors is a crucial route to achieve carbon emission reductions. On the one hand, free exchanges of factors between regions can decrease R&D costs for enterprises [32] and enhance their innovative capabilities [46]. On the other hand, new technologies can be rapidly propagated and applied through regional cooperation channels, further augmenting the regional carbon reduction effect [47]. In summary, the integration policy can bolster regional economic ties, elevate the efficiency of factor flow, and promote carbon reduction.

### 5.3 Enhancement of economic ties: Upgraded digitalization levels

The previous section confirmed that integration policies can enhance inter-regional economic ties and promote inter-regional factor mobility. The knowledge diffusion and technological spillover brought about by factor mobility provide a foundation for regional digital development. Therefore, under the condition of strengthened economic ties, integration will inevitably improve the digital level of provinces. Existing literature indicates that an increase in digital level can enhance the production efficiency of provinces and industries [48], reduce inefficient resource consumption, and consequently decrease carbon emissions [49].

In this study, drawing on the research of Li Haihai et al. [50], the regional digital development comprehensive index was constructed from four aspects: digitalization prevalence, digital device application levels, digital industry investments, and digital industry outputs. Table 7 displays the variables that measure the digital development comprehensive index. The digitalization level was measured using principal component analysis. A higher digital index value indicates a higher level of digitalization. Considering the availability of key indicator data, the sample range spans from 2011 to 2019.

**Table 7:** Digital level indicator system.

Primary Indicator	Secondary Indicator	Indicator Explanation
Digitalization Level	Digitalization Prevalence Represented	Represented by the number of broadband Internet users per hundred people
	Digital Device Application Level	Represented by the mobile phone penetration rate
	Digital Industry Input	Represented by the ratio of employment in the information transmission, software and information technology service industry to the total employment
	Digital Industry Output	Represented by the per capita telecommunication service volume

To examine whether regional integration policies can affect the efficiency of resource use in the region, the dependent variable in Eq (1) is replaced with the variable representing the digital development level of the provinces. The regression results are shown in Table 8, Model (3).

**Table 8:** Mechanism analysis regression results I.

	(1)	(2)	(3)
DID	-0.272***	304.194*	0.076*
	(-2.78)	(1.80)	(1.72)
Control Variables	Y	Y	Y
Time FE	Y	Y	Y
Province FE	Y	Y	Y
N	330	330	270
R^2^	0.808	0.974	0.986
Adj.R^2^	0.775	0.970	0.983

The regression results show that the coefficient of the core variable DID is positive and significant at the 10% level. This indicates that regional integration can promote market integration and factor mobility, utilize knowledge diffusion, and technology spillover effects to enhance the digital development level of provinces, thereby reducing resource consumption and lowering carbon emissions [51].

### 5.4 Provincial industrial structure upgrade: Improved energy efficiency

Carbon emissions mainly stem from energy activities, and a decline in energy consumption leads to a decrease in carbon emissions [52]. The integration policy, while promoting factor mobility, also encourages adjustments in the industrial structure of provinces, creating conditions for the optimization and upgrade of the industrial structure. The elevation of the digital development level in provinces further boosts this industrial structure upgrade. An optimized industrial structure can improve the energy structure of provinces, enhance energy usage efficiency, and thereby amplify the effect of carbon emission reduction [53].

Drawing on the research by Yuan Hang [54], this study measures the upgrade of the industrial structure from two dimensions: the supererogation and the rationality of the industrial structure. The sophistication of the industrial structure encompasses both its magnitude and quality. A higher value for industrial structure sophistication indicates a more advanced structure, whereas a smaller value for industrial structure rationality denotes a more balanced and appropriate structure.

The formula to calculate the magnitude of industrial structure sophistication is:

$$ais1_{i,t}=\sum_{m=1}^{3}\;y_{i,m,t}\times m,\;m=1,2,3$$ (11)

The qualitative calculation formula for the upgrading of the industrial structure is:

$$ais2_{i,t}=\sum_{m=1}^{3}\;y_{i,m,t}\times lp_{i,m,t},\;m=1,2,3$$ (12)

$$lp_{i,m,t}=Y_{i,m,t}/L_{i,m,t}$$ (13)

The calculation formula for the rationalization of industrial structure is:

$${\mathrm{theil}}_{i,t}=\sum_{m=1}^{3}\;y_{i,m,t}ln\left(\frac{y_{i,m,t}}{l_{i,m,t}}\right),\;m=1,2,3$$ (14)

Where: *y*_*i*,*m*,*t*_ represents the proportion of the added value of industry *m* in region *i* during period *t* to the GDP. *lp*_*i*,*m*,*t*_ denotes the labor productivity of industry *m* in region *i* during period *t*. *Y*_*i*,*m*,*t*_ signifies the added value of industry *m* in region *i* at time *t*. *L*_*i*,*m*,*t*_ stands for the number of employees in industry *m* in region *i* during period *t*. *l*_*i*,*m*,*t*_ indicates the proportion of employees in industry *m* in region *i* during period *t* to the total employment.

Due to the inconsistency in the units of measurement between labor productivity and the proportion of industry-added value to regional GDP, this paper employs a centralization method to neutralize the dimensions.

To test whether integration policies can promote industrial structure upgrades and enhance energy-use efficiency, the dependent variable in Eq (1) is substituted with the industrial structure upgrade variable for regression. The regression results are presented in Table 9. Models (1), (2), and (3) in Table 9 correspond to the magnitude, and quality of industrial structure sophistication and industrial structure rationality, respectively. The regression outcomes show that the core variable, DID, has regression coefficients of 0.015, 6.601, and -3.657, significant at the 1%, 10%, and 5% levels respectively. It can be inferred that integration policies, by facilitating factor flows and leveraging regional digital transformation, drive the transformation and upgrade of province industrial structures, thereby enhancing energy efficiency and reducing carbon emissions.

**Table 9:** Regression results for mechanism analysis II.

	(1)	(2)	(3)
DID	0.015***	6.601*	-3.657**
	(2.74)	(1.87)	(-2.31)
Control Variables	Y	Y	Y
Time FE	Y	Y	Y
Province FE	Y	Y	Y
N	330	330	330
R^2^	0.976	0.230	0.876
Adj.R^2^	0.972	0.096	0.854

### 5.5 Mechanism through which regional integration constrains carbon emission efficiency

The basic regression results in the previous sections show that the regional integration policy has suppressed the improvement of carbon emission efficiency in the provinces of the Yangtze River Economic Belt, which is contrary to the expected results. To further verify the underlying mechanisms, this study utilizes input-output data from 2009–2019 of 30 provinces and autonomous regions in China to decompose the *ML* index.

The decomposition formula for the ML index is as follows:

$$ML_{t}^{t+1}={EC}_{t}^{t+1}\times {TC}_{t}^{t+1}$$ (15)

Where, *EC* represents the technical efficiency index, characterizing the ability to achieve maximum output at the current technology level. Given unchanged inputs, optimizing resource allocation will enhance technical efficiency, thereby increasing output. To some extent, this index can measure the region’s ability to allocate resources. *TC* stands for the technological progress index, which represents the degree of output growth due to technological advancements.

To examine why the regional integration policy might reduce the carbon emission efficiency of provinces, we replaced the dependent variable in Eq (1) with the technological progress index and technical efficiency index. The regression results are presented in Table 10.

**Table 10:** Decomposition results for carbon emission efficiency.

	(1)	(2)
DID	0.002	-0.022***
	(0.74)	(-3.73)
Control Variables	Y	Y
Time FE	Y	Y
Province FE	Y	Y
N	330	330
R^2^	0.561	0.431
Adj.R^2^	0.484	0.331

The results in Table 10, model (2), passed the significance test at the 1% level, indicating that the regional integration policy significantly reduced the technical efficiency index (EC) of the provinces in the Yangtze River Economic Belt. This, to some extent, suggests that the implementation of the integration policy has weakened the comprehensive resource allocation capability of regional local governments. According to Table 2, the input factors for carbon emission efficiency mainly include capital, labor, and energy. To further investigate the impact of the integration policy on the resource utilization of provinces in the Yangtze River Economic Belt, this study regresses these three input factors as dependent variables. The results are presented in Table 11.

**Table 11:** Impact of regional integration policy on factor inputs.

	(1)	(2)	(3)
DID	42.697	-327.978	1.57e+08***
	(1.53)	(-1.28)	(4.40)
Time FE	Y	Y	Y
Province FE	Y	Y	Y
N	330	330	330
R^2^	0.995	0.983	0.958
Adj.R^2^	0.994	0.981	0.952

Models (1), (2), and (3) in Table 11 examine the impact of the integration policy on employment, energy, and capital, respectively. The results show that only the coefficient in model (3) is significantly positive, indicating that the integration policy has significantly increased capital inflow. In the short term, it has increased the capital stock of the Yangtze River Economic Belt, but has no significant impact on employment and energy input.

In summary, the integration policy has attracted a substantial influx of capital to the Yangtze River Economic Belt in the short term. However, local governments have not been able to allocate this capital effectively. This may have led to misallocation of capital to regions or sectors with lower energy efficiency, thereby suppressing, to some extent, the improvement of carbon emission efficiency in the Yangtze River Economic Belt.

## 6. Heterogeneity analysis

The empirical study above indicates that regional integration policies significantly reduced the total amount and intensity of carbon emissions in provinces, but did not enhance carbon emission efficiency. The reason lies in the disparities among provinces of the Yangtze River Economic Belt in terms of geographical location, economic development level, and infrastructure construction. This led to different effects of regional integration policies on carbon emissions in different provinces. Below, we analyze the effects on carbon emissions based on economic development levels and geographical location.

In this study, the provinces in the Yangtze River Economic Belt are divided into three tiers based on their levels of economic development: less developed, moderately developed, and highly developed provinces. Moreover, based on the spatial layout of "one axis, two wings, three poles, and multiple points" proposed by the "Yangtze River Economic Belt Development Plan Outline", provinces are categorized into central and peripheral provinces to test the heterogeneous effects of the integration policy. The central provinces are located in the three major growth poles (the growth poles being the Yangtze River Delta provinces, the middle reaches of the Yangtze River provinces, and the Chengdu-Chongqing province group) which include Shanghai, Jiangsu, Zhejiang, Hunan, and other provinces. Peripheral provinces include all other provinces in the Yangtze River Economic Belt that are not central provinces, such as Yunnan, Guizhou, etc. Tables 12–14 present the heterogeneous estimation results for total carbon emissions, intensity, and efficiency.

**Table 12:** Heterogeneity in total carbon emissions.

	(1)	(2)	(3)	(4)	(5)
DID	-75.729***	-35.591**	-65.264***	-52.382***	-70.969***
	(-4.40)	(-2.49)	(-3.13)	(-3.92)	(-3.70)
Control Variables	Y	Y	Y	Y	Y
Time FE	Y	Y	Y	Y	Y
Province FE	Y	Y	Y	Y	Y
N	253	253	242	297	242
R^2^	0.976	0.975	0.976	0.976	0.975
Adj.R^2^	0.971	0.970	0.971	0.972	0.970

**Table 13:** Heterogeneity in carbon emission intensity.

	(1)	(2)	(3)	(4)	(5)
DID	-189.423	-2.679	6.934	47.703	-387.114***
	(-1.64)	(-0.05)	(0.13)	(1.01)	(-2.88)
Control Variables	Y	Y	Y	Y	Y
Time FE	Y	Y	Y	Y	Y
Province FE	Y	Y	Y	Y	Y
N	253	253	242	297	242
R^2^	0.983	0.989	0.989	0.990	0.983
Adj.R^2^	0.979	0.987	0.987	0.988	0.980

**Table 14:** Heterogeneity in carbon emission efficiency.

	(1)	(2)	(3)	(4)	(5)
DID	-0.021**	-0.016*	-0.025***	-0.018***	-0.023**
	(-2.54)	(-1.88)	(-2.75)	(-2.77)	(-2.47)
Control Variables	Y	Y	Y	Y	Y
Time FE	Y	Y	Y	Y	Y
Province FE	Y	Y	Y	Y	Y
N	253	253	242	297	242
R^2^	0.636	0.622	0.643	0.630	0.634
Adj.R^2^	0.563	0.546	0.570	0.562	0.559

In Tables 12 to 14, Models (1), (2), and (3) represent the less economically developed provinces, moderately economically developed provinces, and highly economically developed provinces, respectively. Models (4) and (5) represent central and peripheral provinces.

### 6.1 Heterogeneity in total carbon emissions

As shown in Table 12, the effect of regional integration policies on reducing the total amount of carbon emissions is significant in all regions. However, the effect is somewhat weaker for the moderately developed provinces. The reasons for this disparity are twofold: On the one hand, the moderately developed provinces, mostly situated in the middle reaches of the Yangtze River and neighboring the economically developed eastern provinces, have attracted many energy-intensive, low-value-added industries transferred from the eastern provinces. On the other hand, these moderately developed provinces, under considerable economic development pressure, are more focused on economic growth, resulting in insufficient environmental investments. This is particularly evident when they adopt more relaxed environmental policies while taking over transferred industries. Additionally, with regional integration still in its convergence phase, the "polarization effect" dominates, where highly developed provinces siphon quality resources from their neighboring provinces, potentially increasing their carbon emissions. Moreover, central provinces have a lesser effect on carbon emission reductions than peripheral provinces, primarily due to their superior infrastructure conditions, which results in relatively lower transportation and logistics costs. Coupled with a higher industrial concentration, these central provinces are more attractive for business relocations, thereby increasing the likelihood of regional carbon emissions.

### 6.2 Heterogeneity in carbon emission intensity and efficiency

Table 13 shows that only the peripheral provinces’ carbon emission intensity passed the significance test at the 1% level, while other regions were not significant. However, from the estimated coefficients, the regional integration policy’s effect on reducing carbon emission intensity is negatively correlated with the level of economic development. As seen from the Table 13, the policy’s dampening effect on carbon emission intensity for economically developed and central provinces is weaker than in other regions. This discrepancy arises because the economically developed and central provinces already have a high development level, so the growth effects that the regional integration policy can bring to them are limited, hence their limited space for a decline in carbon emissions.

From Table 14, it can be seen that the regional integration policy’s dampening effect on carbon emission efficiency for moderately developed and central provinces is relatively weak. Although they both passed significance tests at the levels of 10% and 1% respectively, the absolute values of the estimated coefficients are relatively small. The reason is that since moderately developed provinces face significant economic development pressures and central provinces bear the crucial function of leading regional economic development, both prioritize economic growth over environmental protection. Consequently, their decrease in carbon emission efficiency is smaller than in other regions.

## 7. Conclusion and policy recommendations

### 7.1 Conclusion

Utilizing a panel data set spanning 2009–2019 from 30 Chinese provinces, this study employs the proposition of the "Yangtze River Economic Belt Development Outline" in 2016 as a quasi-natural experiment. Leveraging a difference-in-differences model, we explore the ramifications of the regional integration policy of the Yangtze River Economic Belt on provincial carbon emissions. Our principal findings include:

1. The implementation of the "Yangtze River Economic Belt Development Outline" in 2016 has markedly reduced both the total carbon emissions and carbon emission intensity within the region. Nonetheless, it has also, to an extent, hindered advancements in carbon emission efficiency. These conclusions remain robust even after parallel trend tests and placebo checks.
2. Heterogeneity analysis suggests that due to disparities in economic development and geographical positioning, the effects of the integration policy on carbon emissions differ across provinces. Central and more economically advanced provinces witness a relatively marginal impact on carbon reduction and carbon emission efficiency. In contrast, their carbon emission intensity is less restrained.
3. Mechanism tests reveal that while the integration policy fosters regional environmental governance and bolsters economic ties, it also displays evidence that some provincial governments have inadequately allocated a surge in short-term capital inflows, thus suppressing carbon emission efficiency in certain jurisdictions.

### 7.2 Policy implications

In light of the foregoing analysis, to further the high-quality coordinated development of the Yangtze River Economic Belt, we proffer the following policy implications:

1. In the context of integration, it is imperative for local governments to foster the enhancement and evolution of the regional industrial architecture. Less developed regions should embrace industries that elevate their local industrial fabric, aligned with their unique comparative advantages and tangible development conditions. Moreover, enhancing regional collaboration, expediting the establishment of regional innovation ecosystems, and adopting innovation-led strategies are critical. These efforts should aim to expedite the creation of industrial chains characterized by high-added value, mutual benefits, and cohesive upstream connections within the Yangtze River Economic Belt.
2. To dismantle regional barriers, local administrations must propel the development of a cohesive market framework. This entails instituting a standardized market entry protocol, diminishing local governmental interference in the market of factors, eradicating impediments to factor mobility, and fostering the communal sharing of resources and advantages. Furthermore, local governments should augment infrastructure development, enhance inter-regional transportation links, advocate for the uniform provision of public services, and mitigate disparities in regional development.
3. Enhancing the oversight and coordination of capital utilization, local authorities are tasked with augmenting the efficacy of capital distribution. This includes enacting policies designed to channel capital towards less affluent areas, bolster green financial services, and financially underpin businesses’ eco-friendly evolution and growth. Additionally, it is essential to establish a framework for the supervision and coordination of capital to circumvent its underutilization and inefficiency and to refine capital distribution efficiency via competitive mechanisms.
4. By capitalizing on the inherent regional resources, local governments must quicken the pace of technological innovation and digital transformation. Local authorities should assume a pivotal role, refine the industrial configuration, and, through strategic direction, encourage the agglomeration of industries. Enhancing the exchange of information and collaboration among enterprises and effectively amalgamating diverse resources are crucial steps. Moreover, there should be a concerted effort to broaden the application range of technological innovations, persistently advance technological research and development capabilities, and continuously elevate local businesses’ overall digital infrastructure efficiency.

## Supporting information

S1 Data (XLSX)
